# Role of Hematological Parameters in the Grading of COVID-19 and a Model to Predict the Outcome in Inpatients

**DOI:** 10.7759/cureus.45276

**Published:** 2023-09-14

**Authors:** Yogesh Kumar, Amita Kumari, Tribhuwan Kumar, Kamlesh Jha, Md. Zabihullah

**Affiliations:** 1 Physiology, All India Institute of Medical Sciences, Patna, Patna, IND; 2 Physiology, All India Institute of Medical Sciences, Deoghar, Deoghar, IND

**Keywords:** hematological national early warning score 2 (news2), derived nlr, clinical management parameters, covid-19, neutrophil-to-lymphocyte ratio (nlr)

## Abstract

Introduction

Human coronaviruses, identified in the 1960s, are known culprits of respiratory infections. Classified into alpha, beta, gamma, and delta subgroups, these viruses have the capacity to transition from animal reservoirs to causing severe respiratory ailments in humans. Notable outbreaks like the 2003 severe acute respiratory distress syndrome (SARS) epidemic and the ongoing coronavirus disease 2019 (COVID-19) pandemic underscore the recurring emergence of novel coronaviruses with severe human infection potential. COVID-19, driven by severe acute respiratory syndrome coronavirus 2 (SARS-CoV-2), has rapidly become a leading global cause of severe acute respiratory syndrome. Immune system disruptions and cytokine imbalances contribute to severe cases, necessitating early diagnosis and precise severity assessment.

Methodology

This retrospective cross-sectional study encompassed 211 COVID-19 patients admitted to AIIMS Patna from May to July 2020. Clinical and hematological parameters, including neutrophils, eosinophils, basophils, lymphocytes, monocytes, red and white blood cell counts, platelet count, C-reactive protein (CRP), serum ferritin, and d-dimer, were meticulously recorded. Patients were categorized into non-severe and severe groups using the National Early Warning Score (NEWS) 2.

Results

Our findings underscore the pivotal role of hematological markers in gauging COVID-19 severity. Notably, markers such as neutrophil-to-lymphocyte ratio (NLR), derived NLR, lymphocyte monocyte ratio, platelet lymphocyte ratio, d-dimer, CRP, and serum ferritin exhibited notable elevation in severe cases. Survival analysis further established the predictive potential of these markers in assessing disease progression and mortality risk. We advocate for the integration of these markers into existing severity assessment frameworks to foster objective clinical evaluations.

Conclusion

In conclusion, our study unravels the intricate connection between COVID-19 severity and hematological parameters. We emphasize the early warning capabilities of NLR, derived NLR, platelet lymphocyte ratio, and other markers in predicting disease progression. This research underscores the imperative need to incorporate hematological markers into the evaluation of COVID-19 severity, thereby providing invaluable insights for enhancing clinical practice and patient outcomes.

## Introduction

Since their first identification in the 1960s, human coronaviruses have been recognized as causative agents of various respiratory tract infections in humans [1]. These viruses are categorized into four major subgroups: alpha, beta, gamma, and delta, with specific strains known to infect humans. Among the alpha types, 229E and NL63 are common, while OC43, HKU1, SARS-CoV, and MERS-CoV belong to the beta type. Notably, the latter three, including severe acute respiratory syndrome coronavirus 2 (SARS-CoV-2), the most recent addition, have undergone evolutionary shifts from animal coronaviruses to inflict severe respiratory ailments in human populations [2].

Recent history has witnessed three notable outbreaks stemming from coronaviruses: the severe acute respiratory distress syndrome (SARS) outbreak in 2003, the Middle East respiratory syndrome (MERS) outbreak in 2012, and the ongoing coronavirus disease 2019 (COVID-19) pandemic caused by SARS-CoV-2, which emerged in December 2019 [3]. This recurrent pattern indicates the emergence of novel animal-derived coronaviruses with the potential to cause severe human infections at relatively predictable intervals. It is reasonable to anticipate the occurrence of future outbreaks instigated by new strains of coronaviruses.

COVID-19, caused by SARS-CoV-2, has rapidly ascended to become the foremost cause of severe acute respiratory syndrome. This global pandemic has swiftly affected over a million individuals worldwide [4]. Recent research indicates that COVID-19 can result in a wide range of clinical manifestations, ranging from isolated upper respiratory tract issues to severe systemic diseases that impact various body systems, such as the central nervous, gastrointestinal, cardiovascular, immunological, hematopoietic, and reproductive systems, with symptoms varying based on disease severity [5-8].

Presently, it is firmly established that disruption of the immune system and impairment of lymphocytes, notably T-lymphocytes, are frequently observed among patients afflicted with COVID-19 [9]. Additionally, several investigations have highlighted elevated levels of cytokines (TNFα, IL-1, and IL-6) and chemokines (IL-8) in the serum of individuals with severe COVID-19, in contrast to those with milder manifestations, implying a contributory role of hyperinflammatory response in the pathogenesis of the disease [9,10].

In cases where prompt treatment initiation is lacking, affected patients may progress to pneumonia with radiological evidence of parenchymal involvement. The majority of patients (80.9%) present with mild symptoms, whereas a minority experience severe disease (13.8%), and a further 4.7% deteriorate to a critical state [11]. Notably, findings by Huang et al. have indicated elevated plasma levels of proinflammatory cytokines, such as interleukins and tumor necrosis factor-α, among patients admitted to the intensive care unit (ICU), hinting at the potential for cytokine storm effects in severe cases [12]. Given the tendency for many patients to develop acute respiratory distress syndrome within a short time frame after disease onset, there arises a pressing need for the early diagnosis and grading of COVID-19 severity.

Previous research has endeavored to categorize disease severity based on clinical attributes, with the National Early Warning Score (NEWS) serving as one of the most widely accepted scoring systems. Initially introduced by the Royal College of Physicians (RCP) in 2012, the NEWS was devised to enhance the identification, monitoring, and management of hospitalized patients displaying signs of illness [13]. This scoring model relies on a logistic regression framework that predicts in-hospital patient mortality within 24 hours based on vital sign observations [13]. While the original version encompassed pulse rate, respiratory rate, blood pressure, temperature, and oxygen saturation, the 2017 update, NEWS2, incorporated parameters like new-onset confusion and a distinct scoring system for oxygen saturation in patients with type 2 respiratory failure. However, an inherent limitation of this scoring system lies in the subjectivity introduced by the assessor.

To surmount this challenge, the pursuit of a more objective measure becomes imperative. A compelling objective parameter lies in the realm of hematological abnormalities encountered across various coronavirus infections. These abnormalities hold implications for disease progression, severity, and mortality in the context of COVID-19. One such objective measure is the neutrophil-to-lymphocyte ratio (NLR), a marker employed to assess an individual's inflammatory status. Calculated easily from a standard complete blood count, the NLR has previously demonstrated its predictive utility across diverse conditions, including various malignancies, cardiovascular disorders, and rheumatic diseases [14-16].

The platelet count serves as another readily available and straightforward hematological parameter, displaying an independent correlation between disease severity and the risk of mortality within the ICU [17]. Research has illuminated the association between COVID-19 and various coagulopathies, including disseminated intravascular coagulation, venous thromboembolism, sepsis-induced coagulopathy, local microthrombi, and thrombo-inflammation [18].

The response of the human body to COVID-19 is a multifaceted process that exhibits considerable individual variation. While some individuals progress to severe disease, the majority experience milder manifestations, even in instances where exposure to the same infecting strain has occurred. This divergence in outcomes could potentially stem from disparate immunological responses across different individuals. A closer examination of common hematological variables may yield valuable insights into these variations.

The main aim of this study is to investigate the correlation between COVID-19 and hematological parameters. This research seeks to unveil how these parameters may aid in categorizing the severity of COVID-19, offering treating physicians the chance to make informed decisions regarding the most appropriate level of care right at the time of admission.

## Materials and methods

In this retrospective cross-sectional study, we investigated involving patients who were admitted to various COVID wards at AIIMS Patna. Approval for data collection was obtained from the Institutional Ethics Committee ECR/1387/Inst/BR/2020. The study period encompassed three months, specifically from May 2020 to July 2020. A total of 211 patients, admitted in the COVID wards during the study period, spanning a wide age range from twenty to ninety years and encompassing both genders, were included in the study. Patients with pre-existing hematological disorders were excluded from the analysis.

Clinical data, including parameters such as level of consciousness, pulse rate, blood pressure, temperature, respiratory rate, oxygen saturation (SPO_2_), and oxygen flow (if applicable), were collected from patient files. Additionally, hematological parameters were recorded, comprising neutrophils, eosinophils, basophils, lymphocytes, monocytes, total red blood cell (RBC) count, total white blood cell (WBC) count, platelet count, C-reactive protein (CRP), serum ferritin, and d-dimer. The presence of comorbid conditions like diabetes, hypertension, lung disease, thyroid disorders, and heart disease, if applicable, was also documented.

Using the obtained values, we calculated NEWS 2 scores, a widely accepted scoring system. The rationale behind its inclusion lies in its well-established reputation as a standardized and widely accepted clinical tool for assessing illness severity and predicting patient outcomes, particularly in acute care settings. Patients with NEWS scores ranging from 1 to 4 were categorized as non-severe, while those with scores of 5 and above were classified as severe [19].

Statistical analysis

Descriptive analysis was employed for all variables, with data stratified based on disease severity and patient outcomes. Quantitative variables, including WBC, absolute neutrophil count (ANC), absolute eosinophil count (AEC), absolute basophil count (ABC), absolute lymphocyte count (ALC), absolute monocyte count (AMC), and neutrophil-to-lymphocyte ratio (NLR). The NLR is calculated by dividing the number of neutrophils by the number of lymphocytes in a blood sample and derived NLR (the dNLR is a modified version of the NLR that is calculated by dividing the number of neutrophils by the total white blood cell count minus the number of neutrophils), lymphocyte-to-monocyte ratio (LMR), platelet-to-lymphocyte ratio (PLR), hemoglobin (Hb), mean corpuscular volume (MCV), platelet count, ferritin, d-dimer, and CRP were presented as mean and standard deviation.

To compare hematological variables between severe and non-severe cases, as well as between surviving and deceased patients, both the Mann-Whitney U test and Student's t-test were applied. The potential of various variables to predict disease progression was analyzed using Kaplan-Meier curves.

## Results

**Figure 1:**
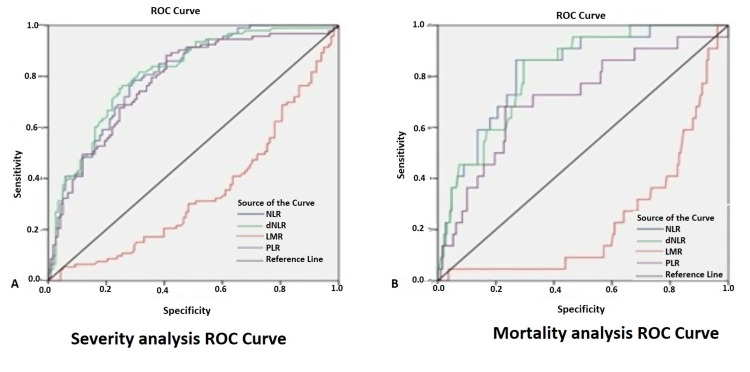
ROC curve showing optimal cut-off points to predict severity and mortality ROC: Receiver operating characteristic; NLR: neutrophil-to-lymphocyte ratio; dNLR: derived neutrophil-to-lymphocyte ratio; PLR: platelet-to-lymphocyte ratio; LMR: lymphocyte-to-monocyte ratio

Data obtained from 211 patients were analyzed. It was observed that 75.35% (n=159) patients were male as compared to 24.6% (n=52) females. Patients with non-severe disease (49.66±12.5) were younger as compared to severe disease (54.24±10.8). Co-morbid conditions were also more common in patients who died or were having severe disease. Table 1 shows a comparison of hematological parameters in severe and non-severe categories. The average severity score in non-severe, severe, survived, and diseased groups is 2.4±1.4, 6.6±1.6, 3.8± 2.5, and 7±2.8, respectively. All markers of immune derangement like NLR, dNLR, LMR, PLR, d-dimer, CRP, and serum ferritin level are significantly increased in the severe category. Table 2 shows a comparison between patients who survived with the diseased subjects. It can easily be observed that in diseased subjects, the NLR, dNLR, and PLR all were very significantly raised at the time of admission itself while LMRs decreased in disease patients. Table 3 and Figure 1 show receiver operating characteristic (ROC) analysis, done to find optimal cut-off points and positive likelihood ratio to predict severity and mortality. To predict mean survival time in days, Kaplan-Meier survival analysis was also done using various variables like NEWS 2 score, NLR, d-NLR, and PLR results which are shown in Table 4.

**Table 1:** Comparison of hematological parameters of COVID-19 patients based on severity (NEWS2 score) ^a^Mann-Whitney U test

Parameters	Overall (n=211)	Non-severe (n=93)	Severe (n=118)	p-value
	Mean (SD)	Mean (SD)	Mean (SD)	
Age (years)	51.7 (12)	49.66 (12. 5)	54.24 (10.8)	0.005
Sex Males	159 (75.3%)	64 (68.82%)	95 (80.51%)	
Females	52 (24.64%)	29 (31.18 %)	23 (19.49 %)	
Hospital stays (days)	11 (6)	10 (5)	12 (9)	0.001^a^
Severity score	4.1 (2.7)	2.2 (1.4)	6. 6 (1.6)	0.001
Absolute neutrophil count	6637.8(5487.3)	5412.9(4229.8)	8375.4 (6008.7)	0.001 a
Absolute eosinophil count	7.9 (59.4)	22.6 (85)	0.0 (15.2)	0.001 a
Absolute basophil count	13.2 (18.3)	13.1 (15.5)	13.4 (18.4)	0.481a
Absolute lymphocytes count	1118 (932.6)	1474.4 (876)	760.9 (590.6)	0.001 a
Absolute monocyte count	223.3 (176.1)	240.1 (154.5)	207.2 (200.9)	0.024a
Neutrophil Lymphocyte Ratio	5.96 (8.7)	3.8 (4.9)	9.4 (12.2)	0.001 a
Derived NLR	4.6 (6.4)	2.96 (3.2)	7.1 (9)	0.001 a
Lymphocyte-Monocyte ratio	4.9 (4.1)	5.7 (5.1)	3.9 (3.1)	0.001 a
Platelet-Lymphocyte ratio	151.1 (166)	103.4 (101)	225.1 (186.1)	0.001 a
Hemoglobin (g/dl)	11.9 (2.1)	12.3 (2.3)	11.8 (3)	0.384 a
Platelet (lakhs/mm3)	1.6 (0.98)	1.5 (0.8)	1.8 (1.1)	0.009 a
RBC (millions/mm3)	4.2 (0.8)	4.2 (0.8)	4.2 (0.8)	0.487
PCV	36.5 (6.1)	36.7 (5.9)	36.3 (6.3)	0.608
MCV	87.5 (6.9)	87.3 (6.2)	87.9 (7.6)	0.514
MCH	28.5 (2.6)	28.4 (2.5)	28.7 (2.9)	0.560
MCHC	32.6 (1.8)	32.5 (1.7)	32.8 (1.8)	0.404^ a^
RDW	14 (1.9)	14 (1.6)	14.1 (2.4)	0.321^ a^
D-dimer	0.77 (1.07)	0.55 (0.7)	1.2 ()1.9	0.001 a
CRP	45.9 (114.6)	26.4 (57.9)	100.9 (139.3)	0.001^ a^
Serum ferritin	447. 5 (618. 5)	324.4 (435.5)	733.5 (894.4)	0.001^ a^

**Table 2:** Comparison of hematological parameters of COVID-19 patients based on the outcome *Mean and SD; ^a^Mann-Whitney U test; NLR: neutrophil-to-lymphocyte ratio; RBC: red blood cell; CRP: C-reactive protein; WBC: white blood cell

Parameters	Survived (n=189)	Deceased (n=22)	p-value
	Mean (SD)	Mean (SD)	
Age (years)	5.8 (11.9)	59.1 (10.7)	0.002
Hospital stays (days)	11 (6)	15 (14)	0.186^a^
Severity score	3.8 (2.5)	7 (2.8)	0.001^a^
Absolute neutrophil count ()	6172.9 (4728.5)	11854.9 (8993)	0.001^a^
Absolute eosinophil count	9.8 (68.9)	0.00 (12.5)	0.006^a^
Absolute basophil count	12.7 (15.6)	25 (26.4)	0.028^a^
Absolute lymphocytes count	1157.5 (1025.4)	693.9 (596.7)	0.001^a^
Absolute monocyte count	223.3 (175.5)	220.5 (187.9)	0.956^a^
NLR	5.4 (7.7)	14.9 (19.1)	0.001^a^
Derived NLR	4.2 (5.7)	10.3 (11.6)	0.001^a^
Lymphocyte-Monocyte ratio	5.2 (4)	3 (2)	0.001^a^
Platelet-Lymphocyte ratio	145 (144.2)	263.7 (249.6)	0.001^a^
Hemoglobin (g/dl)	11.8 (2.1)	12.5 (2.4)	0.001
WBC (/Cmm)	7960 (4615)	12840 (8822.5)	0.001^a^
Platelet (lakhs/mm^3^)	1.58 (0.9)	1.81 (1.2)	0.613^a^
RBC (millions/mm^3^)	4.2 (0.8)	4.3 (0.8)	0.7
D-dimer	0.68 (1)	1.34 (2)	0.005^a^
CRP	44.9 (111.8)	123.1 (169.9)	0.018^a^
Serum ferritin	405.8 (616.6)	765 (994.3)	0.003^a^

**Table 3:** Survival characteristics of COVID-19 patients based on cut-offs of NEWS 2 score, NLR, dNLR, and PLR NLR: Neutrophil-to-lymphocyte ratio; dNLR: derived neutrophil-to-lymphocyte ratio; PLR: platelet-to-lymphocyte ratio

Groups	No of cases	No of events	No censored	Mean survival time in days (95% CI)	Log rank
NEWS 2 score					
< 5	118	2	116 (93.3%)	48 (37, 58)	0.006
≥ 5	93	20	73 (78.5%)	40 (30, 51)	
NLR cut-off 5.86					
< 5.86	104	2	102 (98.1%)	59 (39, 78)	0.012
≥ 5.86	107	20	87 (81.3%)	35 (36, 55)	
dNLR cut-off					
< 4.8	110	2	108 (98.2%)	61 (45, 77)	0.004
≥ 4.8	101	20	81 (80.2%)	34 (27, 41)	
PLR cut-off					
< 177.18	119	6	113 (95%)	50 (33, 67)	
≥ 177.18	92	16	76 (83%)	37 (30, 44)	0.230
NLR cut-off 8.9					
< 8.9	139	3	136 (97.8%)	62 (48, 76)	0.001
≥ 8.9	72	19	53 (73.6%)	32 (25,39)	
dNLR cut-off 6.49					
< 6.49	136	3	133 (97.8%)	62 (48, 76)	0.001
≥ 6.49	75	19	56 (74.7%)	32 (25, 39)	
PLR cut-off 238.7					
< 238.7	152	7	145 (95.4%)	54 (41, 670	0.003
≥ 238.7	59	15	44 (74.6%)	30 (23, 38)	

**Table 4:** Optimal cut-off points of derived parameters for prediction of severity and mortality in COVID-19 patients NLR: neutrophil-to-lymphocyte ratio; dNLR: derived neutrophil-to-lymphocyte ratio; PLR: platelet-to-lymphocyte ratio; LMR: lymphocyte-to-monocyte ratio

Parameters	Optimal cut-offs	Sensitivity	Specificity	Positive likelihood ratio
Severity				
NLR	5.86	0.79	0.71	2.7
dNLR	4.80	0.76	0.75	3.0
LMR	5.81	0.30	0.52	0.6
PLR	177.18	0.68	0.75	2.8
Mortality				
NLR	8.99	0.86	0.73	3.2
dNLR	6.49	0.86	0.70	2.9
LMR	1.87	0.91	0.07	0.9
PLR	238.74	0.68	0.77	2.9

## Discussion

The results of this study illustrate hematological and hemostatic manifestations and their correlation with the severity of the disease in COVID-19 patients. The study reported that males are more affected by the disease than females. A study conducted by Jin et al., reported that according to the clinical classification of severity, men had more severe disease than women [20]. Our study demonstrates that increased NLR, d-NLR, PLR, and decreased LMR which might be due to inflammatory response, have a significant association with the disease severity. The NLR was highest in patients with severe disease. Liao et al. also found an elevated NLR as a useful predictor for the severity and mortality of SARS-CoV2 infection [21]. The association of NLR with the severity of COVID-19 disease was also concurred by a study by Yang et al., who concluded that a high NLR and age are the independent factors for indicating poor clinical outcomes of COVID-19 patients [22]. This study does not show a significant association of platelet count with the severity of disease while Liao et al., found significantly low platelet count in patients with critical and severe disease [21] while Fan et al., found mild thrombocytopenia in 20% of study cases and leukopenia in almost 19% of the total admitted patients, out of which only one person presented with severe leukopenia [22,23]. Lymphopenia was also featured in these patients which was associated with the severity of disease. Hemoglobin, MCV, and hematocrit of the COVID-19 patients showed no association with the severity of the disease in our study. As indicated in Table 5, the test values of NLR, d-NLR, LMR, and PLR on the day of admission provide a basis for determining whether the patient should be admitted to the general ward, high-dependency unit, or ICU.

**Table 5:** Based on the optimal cut-off value of NLR, d-NLR, LMR, and PLR, patients may be graded into non-severe, severe, and critical and accordingly may be admitted to different wards for optimum care NLR: neutrophil-to-lymphocyte ratio; dNLR: derived neutrophil-to-lymphocyte ratio; PLR: platelet-to-lymphocyte ratio; LMR: lymphocyte-to-monocyte ratio

	General Ward Non-severe	High-Dependency Unit (HDU) Severe	Intensive care Unit (ICU) Critical
	Optimal cut-off value		
NLR	< 5.86	5.86 to 8.99	>8.99
d-NLR	<4.80	4.80 to 6.49	>6.49
LMR	>5.81	1.87 to 5.81	<1.87
PLR	<177.18	177.18 to 238.74	> 238.74

Clinical implications and recommendations

Clinical Decision Support

Our findings highlight the potential of hematological parameters, particularly the NLR and other markers, as early indicators of COVID-19 severity. Clinicians can consider these markers as part of their initial assessment to guide decisions on the level of care needed for patients upon admission.

Risk Stratification

Identifying patients at higher risk of severe disease progression is critical. Our study suggests that specific hematological markers can aid in risk stratification. Healthcare providers can use this information to prioritize monitoring and interventions for high-risk patients.

Treatment Tailoring

Understanding the relationship between hematological parameters and COVID-19 severity can help in tailoring treatment approaches. For instance, patients with elevated NLR or other relevant markers may benefit from more aggressive early interventions.

Public Health Measures

Public health authorities can use our findings to emphasize the importance of monitoring and early intervention in patients with specific hematological markers, especially in regions with limited healthcare resources.

Incorporating these clinical implications and recommendations into practice can enhance patient care and contribute to better outcomes in the management of COVID-19.

Limitations of the study

Data Collection Constraints

One limitation of our study was the retrospective nature of data collection, which can introduce inherent biases and incomplete data. Addressing this, we took extensive measures to ensure data accuracy and completeness, but there may still be some residual limitations associated with retrospective data.

Single-Center Study

Another limitation is that our study was conducted at a single medical center, AIIMS Patna. While this allowed for a controlled environment, it may limit the generalizability of our findings to broader populations. Future multi-center studies could provide a more comprehensive perspective.

## Conclusions

In the context of recurring coronavirus infections, effective management of the ongoing pandemic and any potential future outbreaks can be informed by the insights derived from this study. Firstly, the study highlights a gender-based discrepancy in disease outcomes, observing that males tend to face more severe manifestations than females. Secondly, the investigation uncovers compelling connections between disease severity and specific blood parameters. Elevated NLR, dNLR, and PLR, coupled with a reduced LMR, signify heightened inflammation and exhibit a robust correlation with disease severity. Notably, the NLR demonstrates a particularly pronounced elevation among patients with severe disease. Thirdly, this study identifies parameters like hemoglobin, mean corpuscular volume, and hematocrit which do not exhibit significant associations with disease severity. Furthermore, the study proposes the potential utility of easily accessible blood parameters, such as NLR, d-NLR, and PLR, in assigning a severity grade upon patient admission. These parameters offer an avenue for early evaluation, aiding in the prediction of disease severity. The study suggests the practical application of optimal cutoff values for NLR, d-NLR, LMR, and PLR. By categorizing patients into non-severe, severe, and critical groups based on these values, appropriate decisions can be made regarding the allocation of patients to different wards for optimal care (see Table 5 for details.)
